# Evolving the Cybersecurity of Clinical Photography in Plastic Surgery

**DOI:** 10.1055/a-2103-4168

**Published:** 2023-08-02

**Authors:** Daisy L. Spoer, Alexandra Junn, John D. Bovill, Zoë K. Haffner, Andrew I. Abadeer, Stephen B. Baker

**Affiliations:** 1 Department of Plastic Surgery, Georgetown University School of Medicine, Washington, District of Columbia; 2 Department of Plastic and Reconstructive Surgery, MedStar Georgetown University Hospital, Washington, District of Columbia; 3 Division of Plastic Surgery, Michael E. DeBakey Department of Surgery, Baylor College of Medicine, Houston, Texas; 4 Division of Plastic and Reconstructive Surgery, The Warren Alpert Medical School of Brown University, Providence, Rhode Island

**Keywords:** plastic surgery, information technology, Health Insurance Portability and Accountability Act, photography

## Abstract

Point-of-care photography and photo sharing optimize patient outcomes and facilitate remote consultation imperative for resident surgeons. This literature review and external pilot survey study highlight the risks associated with current practices concerning patient privacy and biometric security. In a survey of 30 plastic surgeon residents and attendings, we found that the majority took photos of patients with their iPhones and shared them with colleagues via Apple iMessage. These findings corroborate previous reports and highlight a lack of physician user acceptance of secure photo-sharing platforms. Finally, we frame a successful example from the literature in the context of a postulated framework for institutional change. Prioritizing the privacy and safety of patients requires a strategic approach that preserves the ease and frequency of use of current practices.

Whether to monitor the progression of lower extremity wounds, share “before-and-after” aesthetic photos, or depict intraoperative techniques for a complex surgical case, clinical photography is essential in the practice and progression of plastic surgery. Today, plastic surgeons are equipped with exceptional means for potable capturing, sharing, and storing standardized, high-resolution clinical photographs that improve patient care. 1

Logically, most plastic surgeons use their smartphones to capture (50–90%) and store (46–57%) clinical photography. 1 In a pilot survey of 30 resident and attending plastic surgeons at a single academic institution, we observed that 100% of respondents reported routinely photographing patients (8.2 ± 11.06/d), with their iPhones (80%) and sharing photos via Apple iMessage (67%). These behaviors do not correlate with the intentions of surgeons to protect the sensitive content in photography of patients undergoing breast reconstruction and gender-affirming surgery. Modern facial recognition technology (FRT) adds to the risks of collecting and storing biometric data such as facial features, tattoos, and unique tissue deformities or wounds. These theoretical consequences have surfaced as breaches of plastic surgery photos, and associated biometrics have led to blackmail, ransoms, irreversible identity theft, and permitted access to bank accounts and personal information. 1 2 This is concerning in the context of the Federal Court case, Hazlitt v. Apple Inc., 2021, in which Apple is facing a class action for violating the Illinois Biometric Information Privacy Act. The plaintiffs alleged that the Apple Photos app uses FRT software to collect and store digital faceprint databases that users cannot limit control or remove from their phone. 3

Plastic surgeons face conflicting responsibilities to provide the best possible care for their patients and protect their confidentiality. Merging these duties requires implementing a secure digital tool for point-of-care clinical photography. However, our survey reveals a gap in the institutional and user acceptance of HIPAA-compliant software. Only 57% of plastic surgeons reported having access to an HIPAA-approved method for clinical photo sharing, and 53% cited using the said platform. Marwaha et al present a framework that helps health care organizations (HCOs) navigate institutional and individual barriers to deploying technology, stressing the importance of conducting site-specific needs assessments and interdisciplinary collaboration. 4

Mayo Clinic realized that an outright banning of smartphones was impractical and led to inconsistent behaviors. In response, Mayo Clinic conducted an interdisciplinary review of its local regulations, site-specific policies, institutional framework, and technological bandwidth. 5 This intimate understanding of local workflows and available resources for quality improvement informed their decision to internally develop an iOS-based application, PhotoExam which maintained the convenience of smartphone-based photography while ensuring cybersecurity and privacy for patients. 6 The success at Mayo Clinic suggests that secure point-of-care clinical photography is feasible if HCOs use a strategic approach that respects key considerations consistent with those raised by Marwaha et al. 4 5 6

Ultimately this communication presents the potential consequences of the ongoing widespread disorganization of clinical mobile-photo sharing and offers a solution for individual HCOs to adapt to their unique institutions. The field of plastic surgery and patient confidentiality depends on prioritizing these matters.
